# Examining the Relationship and the Gender Differences between Re-Injury Worry, Confidence, and Attention after a Sport Musculoskeletal Injury

**DOI:** 10.3390/jcm13154428

**Published:** 2024-07-29

**Authors:** Anna Christakou, George Gkiokas, Nikos Valsamis, Eleftherios Paraskevopoulos, Maria Papandreou

**Affiliations:** 1 Lab Biomechanics, Physiotherapy Department, School of Health Sciences, University of Peloponnese, 23100 Sparta, Greece; e.paraskevopoulos@go.uop.gr; 2 Physiotherapy Department, University of West Attica, 12243 Athens, Greece; mpapand@uniwa.gr; 3 Physiotherapy Department, School of Health Sciences, University of Peloponnese, 23100 Sparta, Greece; gkiokasgiorgos12345@gmail.com (G.G.); nickvalsamis02@gmail.com (N.V.)

**Keywords:** re-injury worry, confidence, attention, sport injury, gender

## Abstract

**Introduction**: A sport injury is an unfortunate event that can significantly impact the injured athletes’ psychology. There have been no studies examining gender differences in the psychological state after a sport-related musculoskeletal injury. Τhe aims of the current study were to (a) investigate the relationship between re-injury worry levels, confidence, and attention in the injured athletes who followed a rehabilitation program in order to return to competition, and (b) compare differences in these psychological factors between male and female athletes. **Methods**: A cross-sectional research design was used. A total of 60 athletes (30 men and 30 women) volunteered to participate, aged 25.50 ± 5.25 years old, all of whom had experienced a musculoskeletal injury within the past year. Three validated questionnaires, i.e., the Causes of Re-Injury Worry Questionnaire, the Sport Confidence Questionnaire of Rehabilitated Athletes Returning to Competition, and the Attention Questionnaire of Rehabilitated Athletes Returning to Competition, were completed by the participants on their first competitive game after their rehabilitation program. **Results**: All of the athletes experienced average levels of re-injury worry due to their opponents’ ability and distraction attention, and high levels of confidence and functional attention. Many factors from the questionnaires were correlated with each other. Men experienced lower levels of re-injury worry due to rehabilitation compared to the women athletes (U = 329.00, *p* < 0.05), and women exhibited higher “Re-injury Worry due to Opponent’s Ability” compared to the men athletes (U = 292.00, *p* < 0.01). **Conclusions**: A strong relationship was found between re-injury worry, confidence, and attention, with few gender differences. Since there is no previous research examining re-injury worry, confidence, and attention between men and women, future research with larger and more homogeneous samples is needed to confirm the present results.

## 1. Introduction

Soft tissue sport injuries are almost inevitable in an athlete’s career. Such injuries are unfortunate events that can cause a reduction in training and competition time, leading to decreased sport performance [1]. A previous injury is a strong risk factor for the development of a related re-injury [1,2].

In addition to the physical consequences of an injury or re-injury, such as pain, loss of strength, and diminished functional status, athletes also experience psychological consequences during rehabilitation and upon returning to sport [1,2]. These psychological effects include re-injury worry, decreased confidence, and low levels of attention [1].

Sport worry has four potential content domains, and one of these is the fear of injury [1,2,3]. Higher levels of a fear of injury have been observed in various clinical populations of athletes, including those with a history of patellofemoral pain syndrome, shoulder pathology, low back pain, ankle sprains, and anterior cruciate ligament injuries [4]. Heil’s psychophysiological risk model describes how the fear of a new injury can lead to physical (e.g., muscle tension and hyperarousal) and psychological (e.g., distraction and reduced self-confidence) consequences that negatively affect athletic performance and increase the risk of re-injury [5].

Additionally, worry about re-injury is a predominant emotion as athletes return to sports after completing their rehabilitation program [6]. This constant anxiety is associated with doubt and uncertainty about the possibility of a new injury [7]. Few studies have examined the re-injury worry of previously injured athletes. When athletes do not complete the rehabilitation program or participate in an incorrect program, they may experience re-injury worry [1]. Non-compliance with the rehabilitation program can lead to the improper performance of rehabilitation exercises, resulting in an unhealed injury [1]. An incorrect execution of rehabilitation exercises can decrease coordination and increase muscle tension, both of which are linked to re-injury [1]. Consequently, previously injured athletes who return to their sport may worry about their physical status and may overthink how to execute a skill in detail. This focus on technique can disrupt the flow of movement, potentially leading to re-injury [7].

Another important element related to the psychological state of the athlete upon returning to sport after an injury is self-confidence [8]. Self-confidence is the belief or degree of certainty that a person has during their sporting activity, while injured athlete sport confidence is defined as the belief or degree of certainty about the athlete’s ability to be effective in a skill when returning to competitive activity after an acute musculoskeletal injury [8]. Athletes returning to competitive action after an injury may show hesitancy, which contributes to reduced performance [9]. Doubts about successful performance can create decreased muscle coordination and increased muscle tension, potentially leading to a new injury [7]. According to the stress-injury model, athletes with a history of stressors, such as previous injuries, and personality traits that intensify the stress response, like those experienced during high-demand games or when competing against famous athletes, may exhibit heightened attentional, physiological, and psychological responses. This can include increased muscle tension, which can increase the risk of re-injury. Consequently, negative appraisals of a previous trauma can lead to reduced self-confidence and an increased risk of re-injury during sports competitions. The predisposition to re-injury, as suggested by the stress model, might also impair athletic performance, affecting tactical decision-making or the execution of specific sports skills required in a particular sport. Ultimately, all of these factors, as outlined in the stress model, can lead to reduced self-confidence, diminished attention, and a decrease in sports performance efficiency [10,11].

In the stress-injury model, injury is associated with changes in attentional focus, such as visual field restriction and attentional distraction. The attention of injured athletes is defined as the ability to focus their sensory organs on a source of information, shift focus from one source to another, and track multiple stimuli simultaneously [1,12,13]. When an athlete is under stress, such as during a challenging match, they may not react directly due to peripheral visual restriction, which affects the processing of information. [10,11] The condition of attentional distraction in sports can impact the athlete’s ability to respond appropriately to the rules of the game and the level of participation of the opponent.

Attentional selectivity is one of the key processes for enabling individuals to attend to relevant information and the ability to delete irrelevant and distracting stimuli [14,15]. A selectivity of attention is necessary for effective athletic performance [16], while reduced attention and increased distraction elevate the risk of re-injury and decrease performance [17]. Furthermore, focusing attention on an injured area can cause the athlete to ignore external stimuli in their environment [1,13].

Injuries in contact sports are also studied based on demographic characteristics, particularly on the athlete’s gender. An important issue that arises during the return to competition is the difference between genders. The gender difference in rehabilitation may be due to the difference in biological sex, social expectations, and different situations that men and women face in their sport and everyday lives. The men usually have more muscle mass, strength, and higher testosterone levels compared to women, while women show greater flexibility, less muscle mass, and lower testosterone levels, but have higher estrogen levels, which have been shown to positively affect recovery [18].

Despite the publications regarding the psychological consequences of injuries, little research has evaluated gender differences in previously injured athletes regarding re-injury worry, confidence, and attention before returning to competition. Examining these psychological traits is crucial, as they have a significant role in the rehabilitation of a sport injury [19]. While re-injury worry, confidence, and attention are linked to the quality and effectiveness of the rehabilitation program, no previous studies have investigated any differences between male and female athletes. Understanding these differences could help rehabilitation personnel, such as physiotherapists and coaches, address the specific psychological needs of each sex, thereby improving performance and reducing the risk of re-injury.

Therefore, the aims of the present study were to (a) investigate the relationship between re-injury worry levels, confidence, and attention in the injured athletes who followed the rehabilitation program in order to return to competition, and (b) compare differences in these psychological factors between male and female athletes.

## 2. Materials and Methods

### 2.1. Design

This was a cross-sectional research design. This study has been registered and approved by the Ethics Committee of the School of Health Studies of the University of Peloponnese (201/19 April 2023). The study was in agreement with the Declaration of Helsinki Ethics principles. The outcomes of the study included the completion of three valid questionnaires from the participants.

### 2.2. Participants

The purpose of this study was to investigate, firstly, the re-injury worry, confidence, and attention of previously injured male and female athletes returning to sport, and secondly, any gender differences between these psychological factors using comparison analysis. Thus, we estimated a priori minimum sample size using G-Power analysis. Based on an expected effect size of 0.40 and 80% power (α = 0.05, two-tailed), we aimed to recruit at least 46 athletes. Thirty male and thirty female amateur athletes from different sports, including 18 from soccer, 18 from handball, 7 from basketball, 8 from Taekwondo, and 1 from hockey, voluntarily participated in the present study.

The inclusion criteria for the sample were: (a) being between 18 to 40 years old, (b) participating in contact sports, (c) returning to sport competition after a musculoskeletal sport injury within the last year, and (d) participating in the rehabilitation program.

The mean age of the sample was 25.50 years (SD = 5.25), with a mean competitive experience of 10.06 years (SD = 4.26). All athletes had experienced an acute musculoskeletal injury which included 29 ligament sprains, 13 muscle strains, 10 bone fractures, 6 cartilage tears, and 2 tendon ruptures. The mean duration of the acute musculoskeletal injury was 60.0 ± 7.2 days. The highest frequency of injuries occurred in the lower extremities, particularly at the ankle (24.7%), knee (22.1%), thigh (15.3%), and hip (9.6%). The mean duration of physiotherapy treatment was 42.2 ± 6.8 days, and athletes were absent from sport activities for 54.7 ± 8.6 days. All athletes received medical treatment and completed their rehabilitation program, thus were ready to return to competition (Table 1). Also, in Table 2, we reported the descriptive statistics, frequencies, and percentages between the two genders.

The participants were directly contacted by the 2nd and 3rd authors and informed about (a) the aim of the study, (b) the voluntary nature of participation, and (c) the confidentiality of their responses. Participants registered to the study (convenience sample) and having the inclusion criteria were asked to sign an informed consent document.

### 2.3. Instruments and Procedure

Three valid instruments were used in the study:

(a) The Causes of Re-Injury Worry Questionnaire (CR-IWQ; Cronbach’s α = 0.93), which consists of 2 factors examining 12 questions: “Re-injury worry due to rehabilitation” (Cronbach’s α = 0.95, score range 8–56; higher scores indicate more worry about re-injury due to an ineffective rehabilitation program) and “Re-injury worry due to opponent’s ability” (Cronbach’s α = 0.93, score range 4–28; higher scores indicate more worry about re-injury due to opponent ability) [2].
(b) The Sport Confidence Questionnaire of Rehabilitated Athletes Returning to Competition (SCQ-RARC; Cronbach’s α = 0.92), which consists of 2 factors examining 14 questions: “Confidence due to rehabilitation” (Cronbach’s α = 0.93, score range 8–56; higher scores indicate more confidence due to an effective rehabilitation program) and “General confidence” (Cronbach’s α = 0.97, score range 6–42; higher scores indicate more confidence in one’s own abilities) [8].
(c) The Attention Questionnaire of Rehabilitated Athletes Returning to Competition (AQ-RARC; Cronbach’s α = 0.95), which consists of 2 factors examining 10 questions: “Functional attention” (Cronbach’s α = 0.97, score range 7–49; higher scores indicate higher attention) and “Distraction attention” (Cronbach’s α = 0.94, score range 3–21; higher scores indicate higher distraction) [12].

Additionally, the participants completed a questionnaire with demographical data and the above three questionnaires on the day of their first game after rehabilitation. In particular, the CR-IWQ and the SCQ-RARC were completed before the first game and the AQ-RARC after the first game. The duration to complete the three questionnaires was approximately 15 min.

### 2.4. Statistical Analyses

Descriptive statistics (i.e., means, standard deviations, skewness, and kurtosis), were used for the demographic data and the subscale scores. For the interpretation of the results, mean scores above 61% of the highest score of each factor were considered as high, between 31% and 60% as average, and scores below 30% as low. Kolmogorov–Smirnoff tests were performed to assess normality. Non-parametric analyses, including Spearman’s rank correlation coefficient (Spearman r), were used to analyze the relationships between the variables of the questionnaires. Additionally, Mann–Whitney U tests were conducted to measure any differences between the two genders on re-injury worry, competitive confidence, and attention. We calculated the effect size via r = z/√N (r: effect size; z: z value; N: observation number). We divided z value by the square root of the observation number to obtain the effect size. An effect size r that is less than 0.3 indicates a small effect. An effect size r between 0.3 and 0.5 indicates a medium effect and an effect size greater than 0.5 indicates a large effect [20]. IBM SPSS software Version 23.0 (IBM, Inc., Armonk, NY, USA) was used for all statistical analyses with a statistical significance level set at *p* < 0.05.

## 3. Results

Both men and women exhibited (a) high levels of confidence due to rehabilitation and general confidence, as well as functional attention, and (b) average levels of re-injury worry due to their opponent’s ability and distraction attention. Men displayed lower levels of re-injury worry due to rehabilitation compared to women athletes (Table 3 and Table 4). In Table 5, we reported the correlations among the six factors of the three questionnaires.

The variable “Re-injury worry due to opponent’s ability” showed a statistical difference between women and men (U = 329.00, *p* < 0.05), as did the variable “Confidence due to rehabilitation” (U = 292.00, *p* < 0.01). All of the effect sizes were small, except for the variable “Confidence due to rehabilitation”, which had a medium effect size (Table 6).

## 4. Discussion

The aim of this study was to examine the levels of re-injury worry, confidence, and attention in male and female athletes upon returning to sport after a musculoskeletal injury, and to investigate any gender differences in these psychological variables. Despite the significance of athletes’ psychological state in the rehabilitation process, research assessing gender differences in the psychological state has been lacking. Therefore, it is crucial to examine gender differences in athletes’ psychological characteristics following a sport injury [21].

Both male and female athletes exhibited high levels of confidence due to the completion of their rehabilitation programs and their general confidence. The fact that their rehabilitation was completed successfully suggests that their injuries were likely fully healed. Athletes who are not pressured to return to competition before fully recovering tend to have higher levels of confidence upon their return to sport [7]. Additionally, receiving adequate social support can enhance athletes’ belief in their abilities and help them achieve their goals, thereby boosting their confidence [22]. When the social support athletes receive aligns with their expectations and needs, they tend to have more positive appraisals and fewer negative emotional reactions upon their return to sport. This support also helps them re-evaluate their injury [23].

Furthermore, both men and women exhibited average levels of re-injury worry due to their opponent’s ability. Competing against opponents who appear to play aggressively can trigger negative thoughts in previously injured athletes, such as concerns about getting re-injured due to the high frequency of physical contact with opponents during the game [1].

On the contrary, women appeared with average levels of re-injury worry due to rehabilitation, while men showed low levels. No other studies have specifically assessed the level of re-injury worry among male and female athletes. The quality of the rehabilitation program plays an important role in re-injury worry [1,24]. In this study, it was evident that men were likely to have more confidence in the rehabilitation program and did not worry about experiencing a re-injury compared to women, who felt more concerned about re-injury upon re-entering competition. Additionally, a poor connection with the rehabilitation team could contribute to performing rehabilitation exercises having low adherence. These factors could explain the increased re-injury worry among female athletes, as shown in Table 6. Athletes’ social support from coaches was positively associated with higher self-esteem and vitality levels, which reduced concerns about their return to sport and worries about re-injury [23].

Finally, all participants had average levels of distraction attention and high levels of functional attention. When athletes return to competition with a fully healed injury, they are not thinking of any internal cues from the previously injured area of their body and focus on relevant cues during the game [24]. Additionally, when an athlete receives social support from family members or teammates during the rehabilitation process, this may help them to ignore any negative thoughts regarding their injury, resulting in a higher level of attentional focus and reduced susceptibility to distraction [24].

The overall score of the Causes of Re-Injury Worry Questionnaire was positively correlated with both the causes of re-injury worry due to rehabilitation and the causes of re-injury worry due to their opponent’s ability. The non-return to pre-injury competition levels and the interval between injury and surgery [3] are the main causes of re-injury worry. Other possible causes include the inability to complete the rehabilitation program, pressure on the athlete to return to competitive sports, the lack of social support, the disruption of daily routine practice, pain, and facing a difficult opponent [25,26]. These results align with findings from Jin et al. [27], who reported that athletes participating in sports involving potential contact with opponents (such as football, basketball, handball, and lacrosse) are more likely to experience fear during quick responses to opponents. Additionally, the present results are supported by Christakou et al. [2], who found a positive correlation between the aforementioned factors. Worry about re-injury may affect sports performance and increase the risk of injury. It can lead to both physiological and psychological consequences, which can, in turn, impact sports performance and elevate the risk of injury. An athlete who is concerned about sustaining an injury may perform poorly due to decreased efficiency in the biomechanics of skill execution, reduced self-confidence, and diminished attention [1].

Confidence due to rehabilitation exhibited a positive correlation with general confidence, the overall score of the Sport Confidence Questionnaire of Rehabilitated Athletes Returning to Competition, and the overall score of the Attention Questionnaire, while showing a negative correlation with distraction attention. Decreased confidence can lead to decreased distraction attention [23]. Gkikopoulos et al. [24] reported a high positive correlation among the factors of confidence, rehabilitation confidence, and general confidence, a finding that was also confirmed by the results of the present study.

Confidence due to rehabilitation exhibited a negative correlation with the causes of re-injury worry due to rehabilitation, the causes of re-injury worry due to their opponent’s ability, and the overall score of the Causes of Re-Injury Worry Questionnaire. One of the primary objectives for athletes upon returning to competitive action is to regain confidence in the injured area. For instance, a football player with an anterior cruciate ligament rupture may struggle to perform effectively with the affected body part despite completing rehabilitation successfully. The athlete may also exhibit reduced confidence in their ability during their first competitive game after the injury, resorting to increased reliance on the uninjured limb. Consequently, this may lead to decreased performance and an elevated risk of sustaining another injury. These findings align with the results of Paterno et al. [28], indicating that reduced confidence heightens the risk of knee re-injury. Gkikopoulos et al. [24] emphasized the crucial role of confidence in an athlete’s ability to manage potential complications associated with their injury, while Christakou et al. [12] demonstrated that reduced confidence in an athlete returning to competitive action after a musculoskeletal injury can predict re-injury. Paterno et al. [28] also reported that individuals returning from an anterior cruciate ligament injury with increased confidence due to rehabilitation experienced less re-injury worry. Similarly, Webster et al. [29] argued that athletes with injuries tend to have lower levels of athletic confidence compared to uninjured athletes, and an athlete’s confidence in their ability to perform well is diminished upon returning to sports after an injury. Future studies are warranted to explore the influence of patient personality traits and personal beliefs on athlete confidence and concerns post-injury.

Functional attention exhibited a negative correlation, while distraction attention showed a positive correlation with the causes of re-injury worry due to rehabilitation and the overall score of the Causes of Re-Injury Worry Questionnaire. Attention among rehabilitated athletes is a crucial factor in preventing future musculoskeletal injuries and facilitating faster and better recovery [13]. These findings align with the study by Ohji et al. [30], which suggests that athletes in contact sports may experience fear due to the presence of opponents, leading to increased anxiety about re-injury and the subsequent distraction of attention. Similarly, the present results corroborate the findings of Christakou et al. [1], indicating that re-injury worry may induce psychological changes (e.g., functional attention), which contribute to reduced athletic performance and an increased risk of re-injury. Future research should be conducted to validate the relationship between functional attention, re-injury worry, and the likelihood of re-injury.

Reduced attention may indeed be correlated with re-injury risk when a previously injured athlete returns to competitive action. Jin et al. [31] demonstrated that increased attention helps stabilize the trunk in athletes with a history of anterior cruciate ligament rupture. This suggests that heightened attentional distraction may increase the risk of re-injury in athletes with a history of ligament rupture. Attention plays a vital role in enabling athletes to better control their core during specialized movements required in contact sports. According to Abernethy [14], athletes with a high attention span learn new motor skills more easily and quickly, leading to overall performance improvements. Therefore, by maintaining an adequate level of attention, athletes can enhance their performance, potentially reducing re-injury worry. Further studies investigating the relationship between distraction attention and athletes’ performance are needed to confirm this hypothesis.

Additionally, distraction attention exhibited a positive correlation with the total score of the Attention Questionnaire and a negative correlation with the total score of the Sport Confidence Questionnaire of Rehabilitated Athletes Returning to Competition. These findings corroborate Christakou et al.’s study [32], which identified a correlation between (a) low confidence and distraction attention and (b) high confidence and functional attention. When an athlete returns to competitive action physically and psychologically prepared, it may result in increased confidence, potentially leading to lower distraction attention.

A difference was found between genders in the factor of causes of re-injury worry due to an opponent’s ability and confidence due to rehabilitation. Specifically, women exhibited higher rates of causes of re-injury worry due to their opponent’s ability compared to men, alongside higher rates of confidence due to rehabilitation. While women felt more confident than men regarding the quality of the rehabilitation program, they appeared to worry more about the status of their opponents’ ability during competitive games. Understanding how psychological status influences psychological readiness to return to sport throughout the injury rehabilitation process is crucial [33]. Personality traits are believed to not only influence the recovery process but also predict the outcome of the injury [34]. However, this result is not supported by Ohji et al. [30], who found that both sexes experienced a fear of performing movements or activities due to the possibility of re-injury. This discrepancy might be explained by women experiencing greater daily fluctuations in mood compared to men, associated with improvements or setbacks in their recovery process [35]. Conversely, Lochbaum et al. [36] and Lisse [35] reported different results, stating that men have greater confidence than women. It is possible that gender is not directly linked to the psychological factors examined; rather, these factors may be associated with the individual characteristics of each athlete, irrespective of gender. Therefore, further research is needed to explore how gender affects the psychological state after a sports injury.

In addition to the previously mentioned factors, it is crucial to consider injury-related aspects that may impact re-injury worry among athletes. Variables such as the severity of the initial injury, the specific causes and mechanisms of injury, and the psychological impact of the injury play significant roles contributing an athlete’s belief about re-injury [37]. Severe injuries, for instance, may lead to prolonged recovery times and greater apprehension about returning to play [37]. Understanding the mechanisms underlining an injury can also lead to more effective prevention strategies and rehabilitation protocols, potentially reducing re-injury worry.

Moreover, competitive level may also affect re-injury worry and confidence levels since more advanced methods of rehabilitation may be introduced in elite athletes. Although similar findings have not been reported in the past [38], the limited number of studies available create more uncertainty in this matter.

### Limitations and Future Recommendations

This study has some limitations. Firstly, the participants exhibited heterogeneity in terms of demographic characteristics, such as the severity of injury, duration of abstinence from sports activities, and type of treatment (surgical or conservative), among others. Future studies may examine similar parameters such as re-injury worry and distraction attention in a larger number of athletes with similar sport musculoskeletal injuries that occurred within the last year. Additionally, the level of competition of the sample consisted of non-high-level athletes, which limits the generalizability of the results to all sports categories. High-level athletes may receive more advanced rehabilitation methods, which could influence confidence due to rehabilitation and re-injury worry. Lastly, 18% of the sample did not complete their prescribed physiotherapy program. This subset of patients may exhibit lower compliance and higher stress levels compared to the rest of the sample. These factors could potentially introduce biases and affect the overall results of this study. Understanding the reasons behind their non-compliance and elevated stress could provide valuable insights for future research improving patient outcomes.

Future research should focus on examining the differences in re-injury worry, confidence, and attention among various demographic characteristics. Further studies should explore differences between genders, categories of athletes, and athletes who returned to their pre-injury level and those who did not, as well as differences between those who underwent surgical versus non-surgical treatment with varying lengths of hospital stay. Future investigations could also assess the association between re-injury worry, confidence, and attention in athletes after acute or chronic injury, comparing those who received medication with those who did not. Also, it is important to examine in future research the relationship between the three psychological variables with social support and emotional state.

## 5. Conclusions

Rehabilitated athletes that had a previous injury and return to competition may exhibit higher levels of re-injury worry and distraction attention, alongside decreased confidence. Gender disparities were evident, with men experiencing lower levels of re-injury worry due to rehabilitation compared to women, while women displayed higher levels of re-injury worry due to opponents’ ability. Evaluating these psychological aspects in previously injured athletes is vital for enhancing sports performance and potentially preventing re-injury. Further research is necessary to confirm these gender differences and explore the relationship between medication and athletes’ psychological states. Additionally, future studies should differentiate between various subgroups within the sample, such as amateur versus professional athletes, different injury severities, and surgical versus non-surgical treatment, to provide deeper insights into factors influencing return to sport after injury and inform targeted interventions.

## Figures and Tables

**Table 1:** Frequencies and percentages of the demographic data of the sample (n = 60).

Variables		Frequency f	Percentage Proportion (%)
Sex	Male Female	30 30	50% 50%
Sport	Water polo Martial art Handball Soccer Basketball	3 3 5 21 28	5% 5.1% 8.3% 35% 46.7%
Competitive experience (years)	1–10 10–20 over 20	24 28 8	40% 46.7% 13.3%
Competing level	World European Panhellenic Intercollegiate	1 5 24 30	1.7% 8.3% 40% 50%
Area of injury	Waist Upper limb Lower limb	2 15 43	3.3% 25% 71.7%
Place of injury	Game Training period Leisure time Other	34 17 6 3	56.7% 28.3% 10% 5%
Medical treatment	Conservative Surgical	51 9	85% 15%
Pharmacological treatment	Yes No	27 33	45% 55%
Physiotherapy treatment	Yes	60	100%
Duration of physiotherapy program	1–2 weeks 3–4 weeks 4–5 weeks 6–7 weeks Over 7 weeks	24 17 6 5 8	40% 28.3% 10% 8.3% 13.3%
Number of physiotherapy sessions/week	1 2 3 4 5 6 7	6 14 19 15 1 1 4	10% 23.3% 31.7% 25% 1.7% 1.7% 6.7%
Completion of the physiotherapy program	Yes No	49 11	81.7 18.3

**Table 2:** Descriptive statistics, frequencies and percentages of the demographic data between the genders.

Variables	Male (n = 30)			Female (n = 30)		
Age	Mean SD Range Minimum Maximum Skewness Kurtosis	25.50 years 5.25 years 19 years 19 years 38 years 0.79 −0.22		Mean SD Range Minimum Maximum Skewness Kurtosis	25.57 years 6.63 years 21 years 18 years 39 years 0.81 0.74	
		Frequency f	Percentage Proportion (%)		Frequency f	Percentage Proportion (%)
Sport	Water polo Martial art Basketbal Soccer Handball	- 1 1 15 13	3.3 3.3 49.7 43.3	Water polo Martial art Handball Soccer Basketball Gymnastics	3 2 3 6 15 1	10 6.7 10 20 49.7 3.33
Competitive experience (years)	1–10 10–20 over 20	9 17 4	30 56.7 13.3	1–10 10–20 over 20	15 11 4	50 36.7 13.3
Competing level	World European Panhellenic Intercollegiate	- 2 11 17	- 6.7 36.7 56.7	World European Panhellenic Intercollegiate	1 3 13 13	3.3 10 43.3 43.3
Area of injury	Waist Upper limb Lower limb	1 5 24	3.33 16.7 83.3	Waist Upper limb Lower limb	1 10 19	33.3 33.3 63.37
Place of injury	Game Training period Leisure time Other	20 7 2 1	66.7 23.3 6.7 3.3	Game Training period Leisure time Other	14 10 4 2	46.7 33.3 13.3 6.7
Medical treatment	Conservative Surgical	25 5	83.3 16.7	Conservative Surgical	26 4	86.7 13.3
Pharmacological treatment	Yes No	29 1	96.7 3.3	Yes No	11 19	36.7 63.3
Physiotherapy treatment	Yes	30	100	Yes	30	100
Duration of physiotherapy program	1–2 weeks 3–4 weeks 4–5 weeks 6–7 weeks Over 7 weeks	12 6 4 2 6	40 20 13.3 6.7 20	1–2 weeks 3–4 weeks 4–5 weeks 6–7 weeks Over 7 weeks	12 11 2 3 2	40 36.7 6.7 10.0 6.7
Number of physiotherapy sessions/week	1 2 3 4 5 6 7	1 7 8 10 1 - 3	3.3 23.3 26.7 33.3 3.3 - 10	1 2 3 4 5 6 7	5 7 11 5 1 1 3	16.7 23.3 36.7 16.7 3.3 3.3 10
Completion of the physiotherapy program	Yes No	26 7	86.7 13.3	Yes No	23 7	76.7 23.3

**Table 3:** Descriptive statistics of the questionnaire factors (female athletes).

Questionnaire Factors	Score Range	M	SD	Minimum	Maximum	Skewness	Kurtosis	Percentage *
Overall score on SCQ-RARC	14–98	79.23	12.69	51	98	−0.22	−0.64	-
General confidence	6–42	32.50	6.19	20	42	−0.25	−0.68	77.38
Confidence due to rehabilitation	8–56	46.73	7.50	30	56	−0.48	−0.69	93.85
Overall score on CR-IWQ	12–84	36.63	20.22	12	84	0.57	−0.75	-
Re-injury worry due to opponent’s ability	4–28	14.23	6.12	4	28	0.16	−0.17	50.82
Re-injury worry due to rehabilitation	8–56	22.40	15.79	8	56	0.56	−1.20	40.00
Overall score of AQ-RARC	10–70	47.33	9.45	25	70	0.19	1.13	-
Functional attention	7–49	38.46	8.19	21	49	−0.43	−0.81	78.48
Distraction attention	3–21	8.86	5.34	3	21	0.75	−0.45	42.19

* Percentage of the mean score and the highest value of each factor.

**Table 4:** Descriptive statistics of the questionnaire factors (male athletes).

Questionnaire Factors	Score Range	M	SD	Minimum	Maximum	Skewness	Kurtosis	Percentage *
Overall score on SCQ-RARC	14–98	81.13	2.37	45	98	−1.19	1.51	-
General confidence	6–42	34.13	4.57	26	42	−0.04	−0.94	81.26
Confidence due to rehabilitation	8–56	47.00	9.31	19	56	−1.77	3.53	83.92
Overall score on CR-IWQ	12–84	27.50	14.50	12	63	1.09	0.39	-
Re-injury worry due to opponent’s ability	4–28	11.40	5.41	4	23	0.37	−0.83	40.71
Re-injury worry due to rehabilitation	8–56	16.10	10.81	8	45	1.48	1.29	28.75
Overall score on AQ-RARC	10–70	47.63	6.88	32	58	−0.45	−0.56	-
Functional attention	7–49	40.60	8.10	15	49	−1.17	1.99	82.85
Distraction attention	3–21	7.03	4.67	3	20	1.23	0.81	33.49

* Percentage of the mean score and the highest value of each factor.

**Table 5:** Spearman r correlations among the questionnaire factors.

Questionnaire Factors	Causes of Re-Injury Worry Questionnaire (CR-IWQ)			Sport Confidence Questionnaire of Rehabilitated Athletes Returning to Competition (SCQ-RARC)			Attention Questionnaire of Rehabilitated Athletes Returning to Competition (AQ-RARC)		
	Factors			Factors			Factors		
	1st ^a^	2nd ^b^	Total Questionnaire ^c^	1st ^d^	2nd ^e^	Total Questionnaire ^f^	1st ^g^	2nd ^h^	Total Questionnaire ^i^
CR-IWQ ^a^	0.89 **	0.93 **	1.00	−0.43 **	−0.27 *	−0.41 **	−0.30 *	0.28 *	−0.16
CR-IWR ^b^	1.00	0.72 **	0.89 **	0.47 **	−0.31 *	−0.45 **	−0.31 *	0.28 *	−0.15
CR-IWOA ^c^	0.72 **	1.00	0.93 **	−0.34 **	−0.19	−0.32 *	−0.21	0.21	−0.97
SCQ-RARC ^d^	−0.45 **	−0.32 *	−0.41 **	0.94 **	0.91 **	1.00	0.76 **	−0.06	0.66 **
CDR ^e^	−0.47 **	−0.34 **	−0.43 **	1.00	0.76 **	0.94 **	0.68 **	0.06	0.57 **
GC ^f^	−0.31 *	−0.19	−0.27 *	0.76 **	1.00	0.91 **	0.74 **	−0.05	0.65 **
AQ-RARC ^g^	−0.10	−0.15	−0.16	0.57 **	0.65 **	0.66 **	0.84 **	0.37 **	1.00
DA ^h^	−0.31 *	−0.21	−0.30 *	0.68 **	0.74 **	0.76 **	1.00	−0.17	0.81 **
FA ^i^	0.28 *	0.21	0.28 *	−0.06 *	−0.05	−0.65	−0.17	1.00	0.37 **

^a^ Causes of Re-Injury Worry Questionnaire, ^b^ causes of re-injury worry due to rehabilitation, ^c^ causes of re-injury worry due to opponent’s ability, ^d^ Sport Confidence Questionnaire of Rehabilitated Athletes Returning to Competition, ^e^ confidence due to rehabilitation, ^f^ general confidence, ^g^ Attention Questionnaire of Rehabilitated Athletes Returning to Competition, ^h^ distraction attention, ^i^ functional attention, * *p* < 0.05, ** *p* < 0.01.

**Table 6:** Mann–Whitney U comparisons between genders in questionnaire factors.

Variables	Sex	M Rank	U	Effect Size
CR-IWQ ^a^	Male	26.83	340.00	0.21
	Female	34.17		
Causes of re-injury worry due to rehabilitation	Male	28.18	380.50	0.13
	Female	32.82		
Causes of re-injury worry due to opponent’s ability	Male	26.47	329.00 *	0.23
	Female	34.53		
SCQ-RARC ^b^	Male	32.30	396.00	0.10
	Female	28.70		
Confidence due to rehabilitation	Male	25.23	292.00 **	0.30
	Female	35.77		
General confidence	Male	32.50	390.00	0.11
	Female	28.50		
AQ-RARC ^c^	Male	31.60	417.00	0.06
	Female	29.40		
Distraction attention	Male	27.07	347.00	0.20
	Female	33.93		
Functional attention	Male	33.15	370.00	0.15
	Female	27.85		

^a^ Causes of Re-Injury Worry Questionnaire, ^b^ Sport Confidence Questionnaire of Rehabilitated Athletes Returning to Competition, ^c^ Attention Questionnaire of Rehabilitated Athletes Returning to Competition, * *p* < 0.05, ** *p* < 0.01.

## Data Availability

Data available on request from the corresponding author.

## References

[1] Christakou A., Stavrou N.A., Psychountaki M., Zervas Y. (2022). Re-injury worry, confidence and attention as predictors of a sport re-injury during a competitive season. Res. Sports Med..

[2] Christakou A., Zervas Y., Stavrou N.A., Psychountaki M. (2011). Development and validation of the causes of re-injury worry questionnaire. Psychol. Health Med..

[3] Ardern C.L., Taylor N.F., Feller J.A., Webster K.E. (2012). Fear of re-injury in people who have returned to sport following anterior cruciate ligament reconstruction surgery. J. Sci. Med. Sport.

[4] Barchek A.R., Dlugonski D., Baez S.E., Hoch M.C., Hoch J. (2021). The relationship between injury-related fear and physical activity in people with a history of anterior cruciate ligament reconstruction. Phys. Ther. Sport.

[5] Heil J. (1993). Psychology of Sport Injury.

[6] Haugen E. (2022). Athlete Mental Health & Psychological Impact of Sport Injury. Oper. Tech. Sports Med..

[7] Ardern C.L., Taylor N.F., Feller J.A., Webster K.E. (2013). A systematic review of the psychological factors associated with returning to sport following injury. Br. J. Sports Med..

[8] Christakou A., Stavrou N., Psychountaki M., Zervas Y. (2019). Development and validation of sport confidence questionnaire of rehabilitated athletes returning to competition. Ann. Orthop. Musculoskelet. Disord..

[9] Cheney S., Chiaia T.A., de Mille P., Boyle C., Ling D. (2020). Readiness to Return to Sport After ACL Reconstruction: A Combination of Physical and Psychological Factors. Sports Med. Arthrosc. Rev..

[10] Andersen M.B., Williams J.M. (1988). A model of stress and athletic injury: Prediction and prevention. J. Sport Exerc. Psychol..

[11] Williams J.M., Andersen M.B. (1998). Psychosocial antecedents of sport injury: Review and critique of the stress and injury model. J. Appl. Sport Psychol..

[12] Christakou A., Zervas Y., Psychountaki M., Stavrou N.A. (2012). Development and validation of the attention questionnaire of rehabilitated athletes returning to competition. Psychol. Health Med..

[13] Conti C., di Fronso S., Bertollo M., Robazza C. (2020). A cross-cultural validation of the Attention Questionnaire of Rehabilitated Athletes Returning to competition. Phys. Ther. Sport.

[14] Lee H.I., Park S., Lim J., Chang S.H., Ji J.-H., Lee S., Lee J. (2015). Influence of driver’s career and secondary cognitive task on visual search behavior in driving: A dual-task paradigm. Adv. Phys. Educ..

[15] Memmert D. (2009). Pay attention! A review of visual attentional expertise in sport. Int. Rev. Sport Exerc. Psychol..

[16] Wulf G., Prinz W. (2001). Directing attention to movement effects enhances learning: A review. Psychol. Bull. Rev..

[17] Gray R., Cañal-Bruland R. (2015). Attentional focus, perceived target size, and movement kinematics under performance pressure. Psychol. Bull. Rev..

[18] Nasiakou Μ. (1979). Differences of genders and physiological identity. Greek Rev. Soc. Res..

[19] Slimani M., Bragazzi N.L., Znazen H., Paravlic A., Azaiez F., Tod D. (2018). Psychosocial predictors and psychological prevention of soccer injuries: A systematic review and meta-analysis of the literature. Phys. Ther. Sport.

[20] Cohen J. (1992). A power primer. Psychol. Bull..

[21] Barnett S., Badger G.J., Kiapour A., Yen Y.-M., Henderson R., Freiberger C., Proffen B., Sant N., Trainor B., Fleming B.C. (2020). Females have earlier muscle strength and functional recovery after bridge-enhanced anterior cruciate ligament repair. Tissue Eng. Part A.

[22] Schmitt B., Tim T., McHugh M. (2012). Hamstring injury rehabilitation and prevention of re-injury using lengthened state eccentric training: A new concept. Inter. J. Sports Phys. Ther..

[23] Podlog L., Dimmock J., Miller J. (2011). A review of return to sport concerns following injury rehabilitation: Practitioner strategies for enhancing recovery outcomes. Phys. Ther. Sport.

[24] Gkikopoulos G., Chronopoulou C., Christakou A. (2019). Examining re-injury worry, confidence and attention after a sport musculoskeletal injury. J. Sports Med. Phys. Fit..

[25] Smith A.M. (1996). Psychological impact of injuries in athletes. Sports Med..

[26] Petitpas A., Danish S. (1995). Caring for injured athletes. Sport Psychology Interventions.

[27] Jin P., Ge Z., Fan T. (2022). Team ball sport experience minimizes sex difference in visual attention. Front. Psychol..

[28] Paterno M.V., Flynn K., Thomas S., Schmitt L.C. (2018). Self-reported fear predicts functional performance and second ACL injury after ACL reconstruction and return to sport: A pilot study. Sports Health.

[29] Webster K.E., Feller J.A. (2018). Development and validation of a short version of the anterior cruciate ligament return to sport after injury (ACL-RSI) scale. Orthop. J. Sports Med..

[30] Ohji S., Aizawa J., Hirohata K., Ohmi T., Mitomo S., Koga H., Yagishita K. (2021). Injury-related fear in athletes returning to sports after anterior cruciate ligament reconstruction-a quantitative content analysis of an open-ended questionnaire. Asia Pac. J. Sports Med. Arthrosc. Rehabil. Technol..

[31] Jin P., Zhao Z.-Q., Zhu X.-F. (2023). The relationship between sport types, sex and visual attention as assessed in a multiple object tracking task. Front. Psychol..

[32] Christakou A., Psychountaki M., Stavrou A.N., Zervas Y. Re-injury worry, sport confidence, attention as predictors of re-injuries during a competitive season. Proceedings of the 13th World Congress of Sports Psychology.

[33] Podlog L., Heil J., Schulte S. (2014). Psychosocial factors in sports injury rehabilitation and return to play. Phys. Med. Rehabil. Clin. N. Am..

[34] Cohen R., Baluch B., Duffy L.J. (2018). Personality differences amongst drag racers and archers: Implications for sport injury rehabilitation. J. Exerc. Rehabil..

[35] Lisee C.M., DiSanti J.S., Chan M., Ling J., Erickson K., Shingles M., Kuenze C.M. (2020). Gender Differences in Psychological Responses to Recovery After Anterior Cruciate Ligament Reconstruction Before Return to Sport. J. Athl. Train..

[36] Lochbaum M., Sherburn M., Sisneros C., Cooper S., Lane A.M., Terry P.C. (2022). Revisiting the Self-Confidence and Sport Performance Relationship: A Systematic Review with Meta-Analysis. Int. J. Environ. Res. Public Health.

[37] Covassin T., Beidler E., Ostrowski J., Wallace J. (2015). Psychosocial aspects of rehabilitation in sports. Clin. Sports Med..

[38] Devantier C. (2011). Psychological Predictors of Injury among Professional Soccer Players. Sport Sci. Rev..

